# Bleb Revision using Reversed Scleral Flap and Pedical Conjunctival Graft

**DOI:** 10.5005/jp-journals-10008-1113

**Published:** 2012-08-16

**Authors:** Sourabh Sharma, Dhaval Patel, Reetika Sharma, Tanuj Dada

**Affiliations:** 1 Glaucoma Services, Dr RP Centre for Ophthalmic Sciences, All India Institute of Medical Sciences, New Delhi, India; 2 Glaucoma Services, Dr RP Centre for Ophthalmic Sciences, All India Institute of Medical Sciences, New Delhi, India; 3 Glaucoma Services, Dr RP Centre for Ophthalmic Sciences, All India Institute of Medical Sciences, New Delhi, India; 4 Glaucoma Services, Dr RP Centre for Ophthalmic Sciences, All India Institute of Medical Sciences, New Delhi, India

**Keywords:** Bleb, Conjunctival graft, Hypotony, Trabeculectomy.

## Abstract

Bleb revision for hypotony maculopathy following trabeculectomy is an effective technique for raising intraocular pressure and limiting visual loss. The presence for scleral fistula causing over-filtration obviates the need for reinforcing materials, such as donor sclera or pericardium to cover the defect. However, if the surrounding scleral tissue is healthy, a partial thickness scleral flap can be upturned and sutured over the fistula. Moreover, a vascularized pedical conjunctival graft can also be used in cases where there is a large conjunctival defect.

**How to cite this article:**

Sharma S, Patel D, Sharma R, Dada T. Bleb Revision using Reversed Scleral Flap and Pedical Conjunctival graft. J Current Glau Prac 2012;6(2):94-97.

## INTRODUCTION

The overall success of trabeculectomy with regard to intraocular pressure (IOP) control has increased with the use of mitomycin C (MMC).^1^ However, the associated complication of hypotony maculopathy has also risen with its use. The cause of hypotony after trabeculectomy can be associated with over filtration, wound leak or reduced aqueous production, which may be related to inflammation. In some patients, the hypotony is associated with a maculopathy, which classically consists of variable degrees of fine radiating foveal striae, choroidal folds, tortuous vessels and disk edema.

Many different techniques have been attempted in managing the hypotony resulting from overfiltering blebs, including bandage contact lens^2,3^ Simmons shell, autologous blood injection^4-6^ trichloroacetic acid,^7^ argon^8,9^ or neodymium: YAG laser,^10,11^ compression sutures^10,11^ and cataract surgery.

We report a case of 27-year-old Asian Indian woman who underwent trabeculectomy with MMC for juvenile open angle glaucoma and postoperatively developed hypotonic maculopathy.

**Fig. 1:**
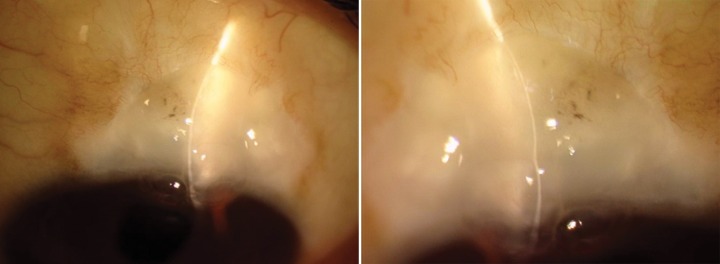
Clinical picture

**Fig. 2:**
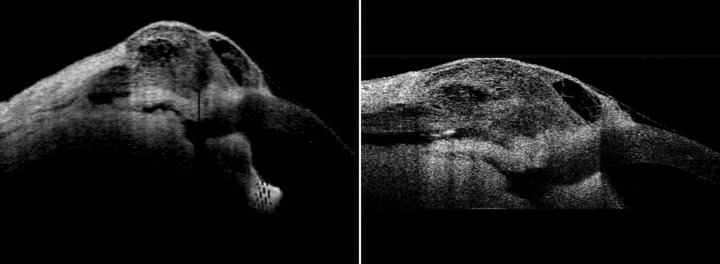
Preoperative ASOCT

She presented with complaint of low vision and pain 6 months after the surgery. On examination, the bleb was localized superiorly, about 3 clock hours and was thin, avascular with cystic changes (Fig. 1). The IOP was 4 mm Hg. The fundus examination revealed foveal striae. The findings were confirmed with ASOCT anterior segment (AS)-OCT (Fig. 2) and macular OCT. The patient underwent bleb revision with conjunctival closure using pedicle conjunctival flap.

### Technique

After prepping the patient, an inferior traction suture was placed. Anterior chamber was entered using micro vitrectomized blade (MVR) and air was injected to form the anterior chamber. The avascular bleb tissue was circumscribed using gentian violet dye and was excised with Vannas scissors, exposing the scleral flap. The underlying scleral tissue was found to be necrosed with fistula formation. A partial thickness sclera flap of about 8 × 4 mm posterior to the necrosed sclera using crescent blade was created. This newly created flap was then upturned and sutured over the necrosed sclera using 10’0 monofilament suture.

**Fig. 3:**
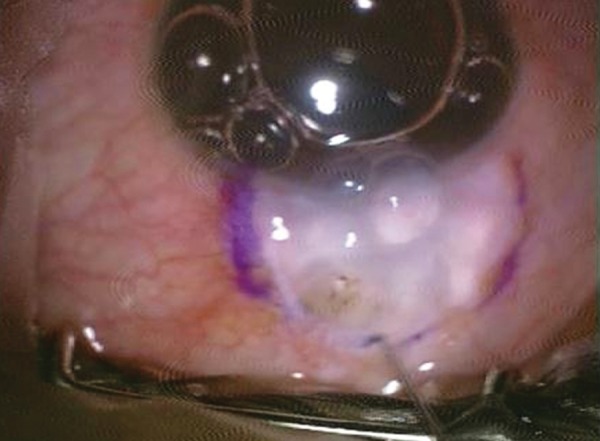
Marking of bleb

**Fig. 4:**
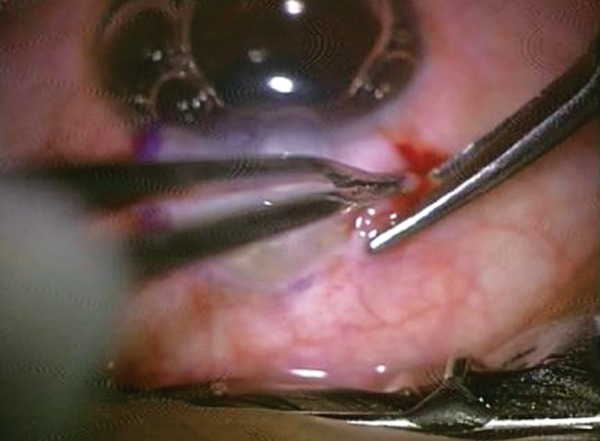
Excision of bleb

**Fig. 5:**
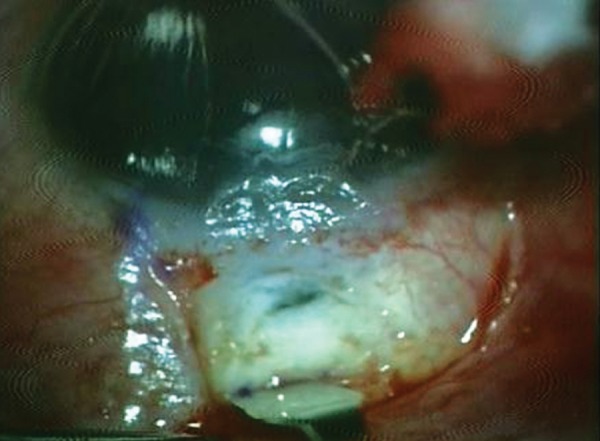
Sclera fistula

**Fig. 6:**
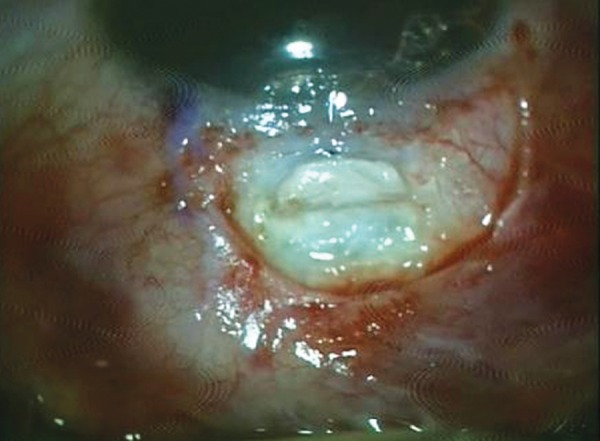
Reversed sclera flap

**Fig. 7:**
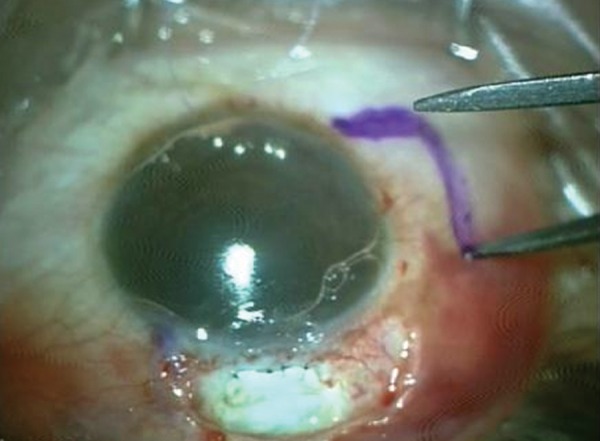
Marking of pedicle flap

**Fig. 8:**
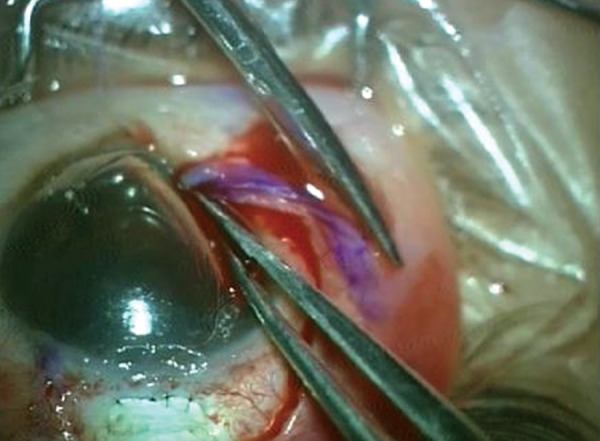
Excision of pedicle flap

**Fig. 9:**
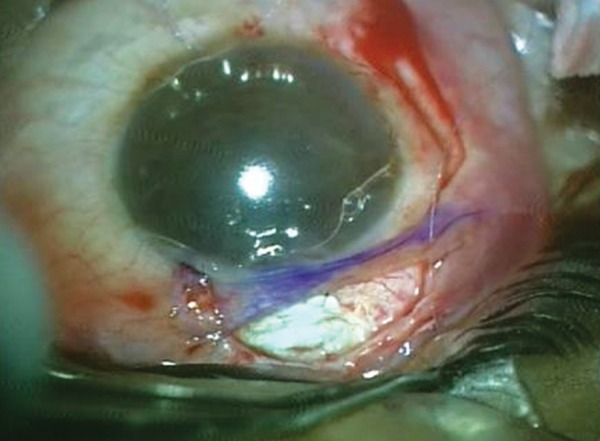
Rotation of flap

**Fig. 10:**
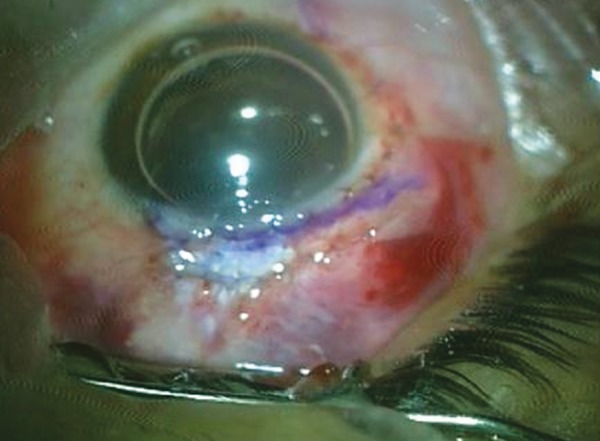
Sutured flap

The conjunctival defect was then measured using calipers and an area of about 20% extra dimensions was marked on the adjoining healthy vascularized conjunctiva leaving 1 mm from the limbus to prevent stem cell deficiency. The conjunctiva was excised using Vannas scissors to create a pedicle flap. It was then rotated and sutured to the conjunctival defect with continuous absorbable vicryl sutures. The donor site was closed by apposition (Figs 3 to 10).

**Fig. 11:**
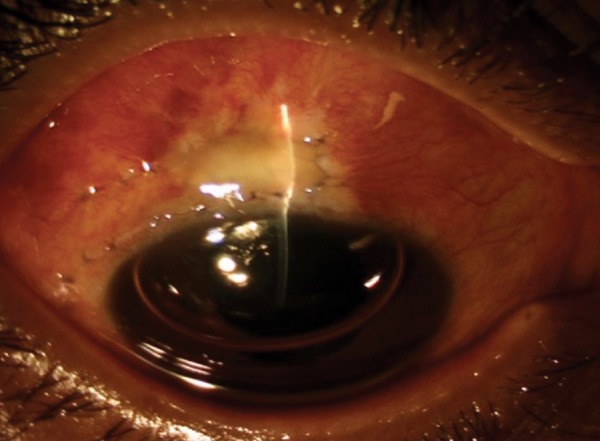
Postoperative slit photograph showing a formed bleb and chamber

**Fig. 12:**
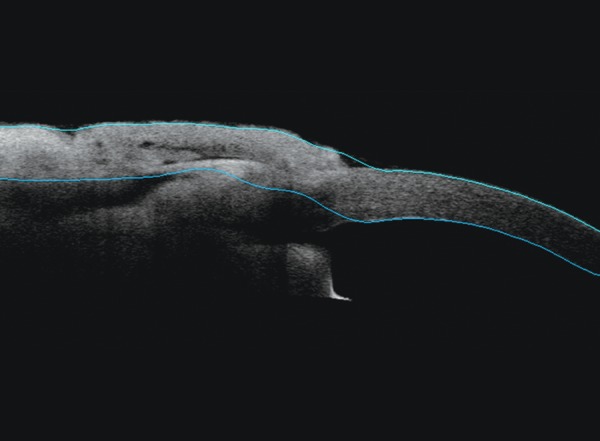
Postoperative ASOCT

On the first postoperative day, the IOP was 6 mm Hg. The wound was healthy with a negative Seidel’s test (Figs 11 and 12). On subsequent follow-ups, the IOP of the patient showed gradual increase to early teens with gain of vision.

## DISCUSSION

With the increased use of the antimetabolite, MMC, as an adjunct to trabeculectomy, hypotony maculopathy has become an increasingly common and serious postoperative complication.^12-14^ Overfiltration is an important factor accounting for the hypotony maculopathy. It occurs when aqueous outflow exceeds aqueous production due to decreased resistance to outflow. Hypotony secondary to scleral fistula as created during trabeculectomy can result in a diffuse, extensive bleb and a low IOP without wound leak. The associated reduced vision can be permanent, if the hypotony is not corrected.

Many different techniques have been attempted in managing the hypotony from overfiltering blebs as well as for the surgical revision of an overfiltering bleb.^15-18^ Where there are obvious direct communications from the anterior chamber visible after conjunctival removal, the filtration site can be reinforced with additional tissue. Donor sclera (split to half thickness), donor pericardium and Tenon’s connective tissue are some of the reinforcing materials which have been used.^18^ In our case, though the scleral necrosis and fistula were seen, the surrounding sclera seemed to be healthy and of adequate thickness. So, we decided to take a partial thickness scleral flap to cover the scleral defect which was the presumed cause of hypotony in our patient.

Closure of the exposed region in bleb repair has also been described by various methods. When the surrounding conjunctiva can be mobilized by blunt dissection, it is advanced to cover the defect. In some cases, the closure can be made by joining the sides of the cut edges.^18^ This however, was not possible in our case as the conjunctival defect was too large, which could have led to ptosis and severe contracture.

A different approach to closure in bleb repair is taken when the surrounding conjunctiva is too immobile to cover the defect. Here, an autologous conjunctival graft from different area of the same eye is used. However, it is seen that some free grafts become extremely thin over years after revision surgery. It may be noted that transplantation of all the subepithelial connective tissues can mitigate this thinning. Nonetheless, the lack of vascular ingrowth into free grafts may predispose to this thinning. For this reason, we preferred closure of the defect by mobilization or rotation of neighboring, vascularized conjunctiva.

## References

[1] Azuara-Blanco A, Katz LJ (1998). Dysfunctional filtering blebs.. Surv Ophthalmol.

[2] Blok MD, Kok JH, van Mil C, Greve EL, Kijlstra A (1990). Use of the Megasoft bandage lens for treatment of complications after trabeculectomy.. Am J Ophthalmol.

[3] Nuyts RM, Greve EL, Geijssen HC, Langerhorst CT (1994). Treatment of hypotonous maculopathy after trabeculectomy with mitomycin C.. Am J Ophthalmol.

[4] Choudhri SA, Herndon LW, Damji KF, Allingham RR, Shields MB (1997). Efficacy of autologous blood injection for treating overfiltering or leaking blebs after glaucoma surgery.. Am J Ophthalmol.

[5] Wise JB (1993). Treatment of chronic postfiltration hypotony by intrableb injection of autologous blood.. Arch Ophthalmol.

[6] Leen MM, Moster MR, Katz LJ, Terebuh AK, Schmidt CM, Spaeth GL (1995). Management of overfiltering and leaking blebs with autologous blood injection.. Arch Ophthalmol.

[7] Gehring JR, Ciccarelli EC (1972). Trichloroacetic acid treatment of filtering blebs following cataract extraction.. Am J Ophthalmol.

[8] Hennis HL, Stewart WC (1992). Use of the argon laser to close filtering bleb sleaks.. Graefes Arch Clin Exp Ophthalmol.

[9] Akova YA, Dursun D, Aydin P, Akbatur H, Duman S (2000). Management of hypotony maculopathy and a large filtering bleb after trabeculectomy with mitomycin C. Success with argon laser therapy.. Ophthalmic Surg Lasers.

[10] Zacchei AC, Palmberg PF, Mendosa A, Robinson JC (1996). Compression sutures: A new treatment for leaking or painful filtering blebs.. Invest Ophthalmol Vis Sci.

[11] Palmberg P, Leader B, Calckwood J (1996). Late complications after glaucoma filtering surgery.. Proceedings of the 45th Annual Symposium of the New Orleans Academy of Ophthalmology..

[12] Costa VP, Wilson RP, Moster MR, Schmidt CM, Gandham S (1993). Hypotony maculopathy following the use of topical mitomycin C in glaucoma filtration surgery.. Ophthalmic Surg.

[13] Zacharia PT, Deppermann SR, Schuman JS (1993). Ocular hypotony after trabeculectomy with mitomycin C.. Am J Ophthalmol.

[14] Suner IJ, Greenfield DS, Miller MP, Nicolela MT, Palmberg PF (1997). Hypotony maculopathy after filtering surgery with mitomycin C. Incident and treatment.. Ophthalmology.

[15] Van de Geijn EJ, Lemij HG, de Vries J, de Waard PW (2002). Surgical revision of filtration blebs: A follow-up study.. J Glaucoma.

[16] Cohen SM, Flynn HW, Palmberg PF, Gass JD, Grajewski AL, Parrish RK 2nd (1995). Treatment of hypotony maculopathy after trabeculectomy.. Ophthalmic Surg Lasers.

[17] Catoira Y, Wudunn D, Cantor LB (2000). Revision of dysfunctional filtering blebs by conjunctival advancement with bleb preservation.. Am J Ophthalmol.

[18] La Borwit SE, Quigley HA, Jampel HD (2000). Bleb reduction and bleb repair after trabeculectomy.. Ophthalmology.

